# Using animations to teach biological processes and principles

**DOI:** 10.1371/journal.pbio.3001875

**Published:** 2022-11-17

**Authors:** Pamela Kalas, Rosemary J. Redfield

**Affiliations:** 1 Department of Botany, University of British Columbia, Vancouver, British Columbia, Canada; 2 Department of Zoology, University of British Columbia, Vancouver, British Columbia, Canada

## Abstract

Well-designed animations can help biology students focus on principles and processes rather than relying on rote memorization. This Community Page highlights question-driven, terminology-free, ‘candymation’ videos that show how and why mitosis and meiosis work the way they do.

## Teaser

Well-designed animations can help students to focus on the underlying principles and processes in biology rather than relying on rote memorization. We present question-driven, terminology-free, “candymation” videos for teaching the concepts behind mitosis and meiosis as an example.

## Biology’s memorization problem

Most students are initially eager to learn biology, but their enthusiasm often turns to disappointment as they struggle to memorize details whose significance has not been explained. This memorization problem begins in high school but persists through the first years of university [1]. For example, students in typical introductory biology courses learn the steps and subcellular locations of photosynthesis reactions, reproduce diagrams of floral reproductive systems and the chambers of the heart, and apply Mendel’s Laws to genetic crosses, but often do not realize that the real excitement in biology arises from the challenges these processes evolved to solve and the elegance of the solutions.

Textbooks are usually the primary resource, both for students and for instructors who lack the luxury of teaching only their areas of personal expertise, but the role of textbooks as learning guides has been swamped by their role as reference books, burying the broad concepts in a morass of details. In principle, animations can help learning by clarifying events and processes that are not easily observed or described in text and static images [2]. Unfortunately, the animations and other resources provided with biology textbooks usually just reinforce the textbooks’ emphasis on facts, and the animations available on sites such as YouTube are no better—whether created by students for class assignments or by non-academic educators, they typically just add movement to the textbook illustrations, focusing on the facts students need to remember rather than the processes and principles we want them to understand.

Most instructors are eager to shift the emphasis from rote memorization to true learning, both in their own classrooms and by providing resources for others to use. Whereas the work entailed in writing a better textbook puts this endeavor out of reach for almost everyone, creating and distributing animations has literally become child’s play, thanks to video hosting sites such as YouTube and inexpensive stop-motion apps. Instructors who want to change what and how their students are learning can now create and share videos that develop curiosity, promote conceptual understanding, and discourage rote memorization.

## Developing animations to teach biological processes

We have focused our efforts on creating concept-driven animations and supporting materials (Box 1) for teaching mitosis and meiosis, processes that students find notoriously difficult. Typically, both textbooks and instructors describe and illustrate the events of mitosis and meiosis as “stages,” an approach that encourages memorization and neglects the challenges that these divisions must solve. Most students respond by learning only the terminology and simple diagrams, and retain this knowledge only long enough to pass their exams. The intrinsic difficulty of the terminology exacerbates the memorization problem: many terms sound similar (e.g., mitosis/meiosis, centromere/centrosome/centriole, chromosome/chromatid/chromatin), and few give students who are not scholars of Greek or Latin any clues to their actual meanings (e.g., kinetochore, telophase, pachytene, chiasmata). Thousands of YouTube and textbook-provided videos teach the stages and the terminology, but watching the top-ranked 300 videos revealed that none of these provide the functional perspective that explains “why” mitosis and meiosis work the way they do. By contrast, our materials make this very explicit to learners; focusing on the “how” and “why” instead of just on the “what” to make that point even more explicit.

**Box 1. Resources for teaching mitosis and meiosis**

**Animations and associated resources we have created**

- Animation: “The Facts of Life: Mitosis” on YouTube and at Useful Genetics
- PDF: “Instructors Guide for Mitosis” at Useful Genetics
- Animation: “The Facts of Life: Meiosis” on YouTube and at Useful Genetics
- PDF: “Instructors Guide for Meiosis” at Useful Genetics
- PowerPoint slide deck: “Pair-pull-part strategy” at Useful Genetics
- Useful Genetics Module 7: Seventeen lecture videos (approx. 10 min each) on mitosis, meiosis and mating, and 2 multiple-choice problem sets at Useful Genetics

**Repositories for educational resources**

- MERLOT (Multimedia Educational Resource for Learning and Online Teaching)
- OER commons (a public digital library of open educational resources)
- The OASIS tool will search a wide range of other open-access repositories

**Other resources we like**

- Video from 1956 of a lily cell mitosis, showing the chromatid arms synapsed through metaphase and then springing apart
- Articles on teaching meiosis [3,4]
- Animation showing the role of tension between kinetochores and fibers in mitosis
- For instructors who want to learn more about meiosis, a 2017 talk by Professor Peter Donelly on YouTube

**Fig 1:**
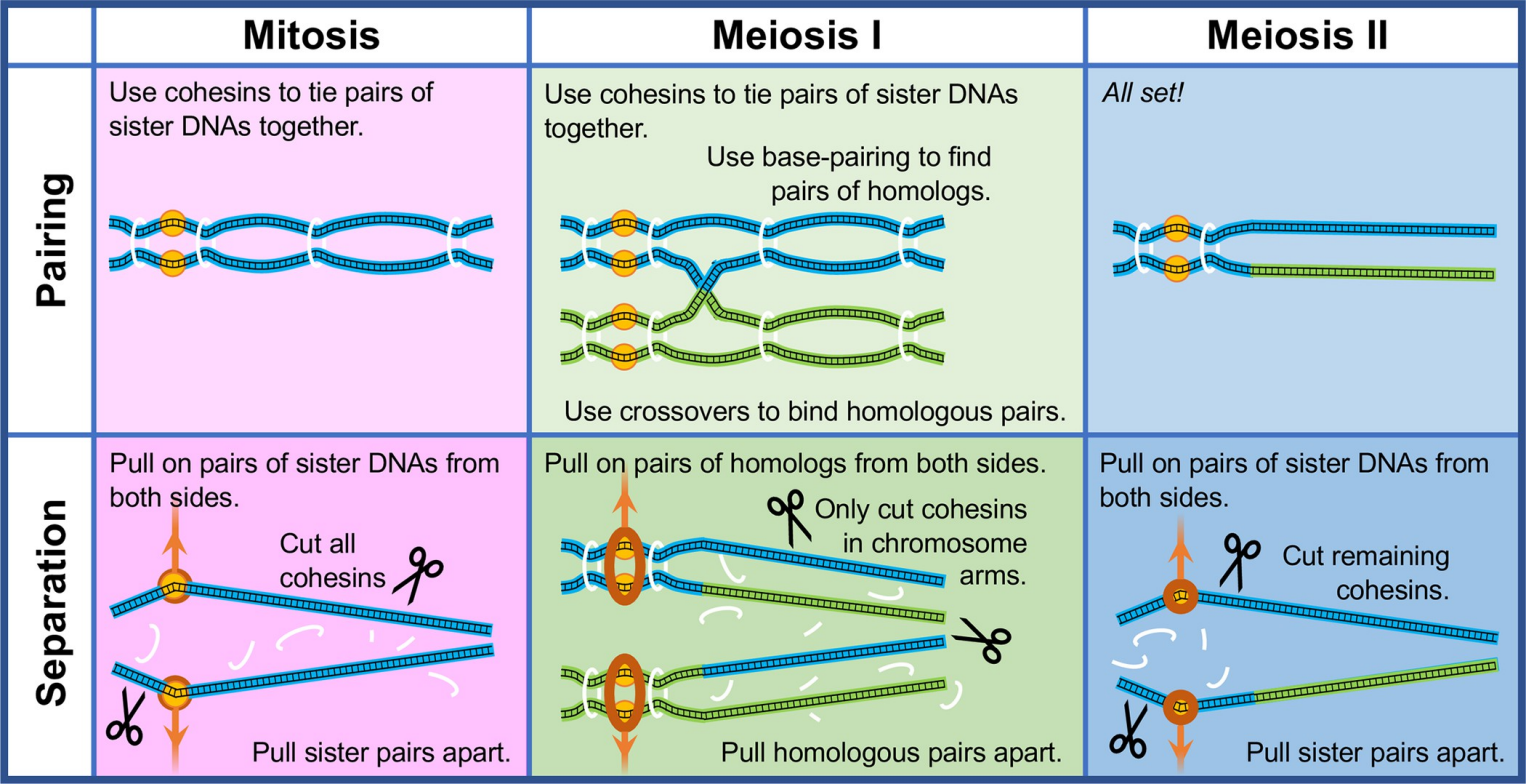
The pair-pull-part strategy shared by mitosis and meiosis. Mitosis and Meiosis II (left and right upper panels) bypass genetic information entirely, instead using the proximity of newly replicated sister chromatids to direct cohesin proteins to tie them together. Meiosis I (center upper panel) identifies homologous chromosomes, not with sequence-recognition proteins, but by their ability to form inter-chromosome base pairs, and uses changes in their physical connectivity (crossovers) to stabilize the pairing. For all 3 divisions, correct orientation for separation is directed by the physical tension on their connection to spindle fibers (middle panels), and the actual separation occurs when the cohesin loops are cut by checkpoint-regulated separase proteins (lower panels). Blue and green lines, chromosomes and chromatids; black lines, DNA; yellow circles, centromeres; orange rings, kinetochores; orange arrows, spindle fibers; white loops, cohesins; scissors, separases.

Mitosis and meiosis are processes that both must solve the same problem: distributing chromosomes so that each daughter cell has a complete set (or sets) of genetic information. The “pair-pull-part” strategy they evolved to do this is rarely made explicit, but provides a conceptual framework that unites the 2 processes and explains their functional logic (Fig 1). Remarkably, this strategy relies mainly on physical information rather that the DNA sequences that distinguish the chromosomes.

Our new animations (created on a Mac using a webcam and the iStopMotion and iMovie apps) present the processes of mitosis and meiosis using a series of “How?” and “Why?” questions, each followed by a visual explanation acted out by candy chromosomes (Box 1). There are no voice-over explanations; students discover the answers by watching what happens, ideally with instructor guidance. For example, instructors could pause the meiosis video at 8:17 to point out the upcoming comparison of the pair-pull-part strategy in all 3 divisions. The components are not labeled, leaving instructors free to decide how much terminology their students need to learn. Molecules are illustrated in ways that emphasize their functions, not their structures—sister chromatids are held together by loops of string (cohesins), fiber-kinetochore attachments are checked by a toy inspector with a clipboard (the spindle assembly checkpoint), and when it is time for the chromatids to separate, the cohesin strings are cut by tiny scissors (separases). The candies used for the components make the videos engaging, and their physical flexibility gives a strong sense of the forces acting on the real molecules.

The animations have been posted on YouTube, on our Useful Genetics site, and at educational resource repositories (Box 1). The shared “pair-pull-part” strategy is also illustrated in a short set of PowerPoint slides. To enable instructors to direct student learning without having to themselves become experts, each animation has an associated Instructors Guide that includes a detailed list of the many questions students might pose at various points in the animation, each with an answer. These questions can be adapted for use in assessments in both open-book and closed-book formats. Since the animations present only the “standard” cases, the guides also address important issues and exceptions, such as why meiosis uses DNA replication followed by 2 divisions, and the lack of meiotic crossing-over in male *Drosophila*.

We hope that these animations and associated resources can help instructors who teach mitosis and meiosis to shift their students’ focus from memorization to understanding. But biology’s memorization problem extends far beyond cell division, and we hope that other instructors, seeing how effective low-tech animations can be, will be inspired to use their pedagogical insights and process-specific expertise to create and share additional resources on other topics. When teaching emphasizes the challenges organisms face and the strategies evolved to solve them, students can find in their classes the same excitement that brought them to biology.
